# Modulation of radiation-induced and mitomycin C-induced chromosome damage by apigenin in human lymphocytes *in vitro*

**DOI:** 10.1093/jrr/rrs117

**Published:** 2013-06-13

**Authors:** Narinder K. Sharma

**Affiliations:** Genetic Toxicology and Chromosome Studies Section, Radiation Biology and Health Sciences Division, Bhabha Atomic Research Centre, Trombay, Mumbai 400085, India

**Keywords:** Radiation, chromosomal aberration, cytochalasin-B blocked micronuclei, sister chromatid exchange, mitomycin C, antimutagenic, apigenin

## Abstract

Apigenin (APG), a flavone, is known to exhibit antioxidant, antimutagenic and antitumorigenic activity, both *in vivo* and *in vitro*. The aim of this study is to investigate the modulatory effects of APG on human lymphocytes after irradiation with gamma rays (3 Gy) or treatment with the antineoplastic agent, mitomycin C (MMC), *in vitro.* Cytogenetic biomarkers such as chromosome aberrations (CAs), sister chromatid exchanges (SCEs) and cytochalasin-B blocked micronuclei (CBMN), were studied in blood lymphocytes treated with radiation, or antineoplastic agent (MMC), and APG. Whole blood lymphocytes were cultured *in vitro* using a standard protocol. No significant differences were found in the frequency of CAs or micronuclei (MN) in human peripheral blood lymphocytes irradiated with gamma rays (3 Gy) and then post-treated with APG. There was an increase in the frequency of SCEs per cell in APG-treated samples compared with the controls. Lymphocytes treated with MMC in the presence of APG exhibited a significant decrease (*P* < 0.01) in the frequency of SCEs compared with MMC treatment alone. The data for the MN test indicated that APG treatment significantly reduced (*P* < 0.01) the frequency of MMC-induced MN.

## INTRODUCTION

One important focus of radiobiological research is to protect living organisms from radiation-induced damage. Ionizing radiation and alkylating compounds commonly used in the therapeutic treatment of malignant diseases, induce different kinds of damage to the cellular macromolecules. Exposure to ionizing radiation potentially increases the risk of adverse health effects. Victims of nuclear fallouts and nuclear terrorism, workers in the nuclear power industry, waste clean-up crews, people living in homes surrounding nuclear plants or research laboratories, patients undergoing routine diagnostic or therapeutic radiation treatment, and members of the armed forces, are potentially subjected to intentional or unintentional sources of radiation [1].

Exposure levels among people working in radiation areas, radiation therapeutics, chemotherapeutic and antineoplastic drugs, vary greatly from small to very high doses, resulting in different health risks. Ionizing radiation induces a number of kinds of damage to cellular macromolecules. Hydroxyl radicals produced by the radiolysis of water withdraw H atoms from the C'-4 position of deoxyribose, contributing to DNA breaks. Natarajan *et al*. showed that radiation-induced double strand breaks (DSBs) are mainly responsible for chromosomal aberrations (CAs). They result in unstable aberrations like dicentric and acentric fragments, and acentric rings [2, 3]. It is an established fact that the biological effects of radiation results are due to energy deposition in irradiated cells causing reactive oxygen species (ROS) to be produced [4]. Several endogenous antioxidant enzymes are capable of scavenging ROS, and repairing DNA damage induced by ROS. However, due to overproduction of ROS, oxidative damage can lead to radiation-induced cytotoxicity or lethality [5, 6].

The potential use of flavonoid compounds as a radioprotector is widely known [7]. Much of the information on the protective effects of flavonoids has come from epidemiological studies suggesting that high fruit and vegetable consumption is associated with a decreased risk of several types of cancer. Flavonoid compounds are known to protect against certain forms of cancer and aging, possibly by preventing initial DNA damage. The protective effects of flavonoids, together with their potent antioxidative and free radical-scavenging activities, have increased the use of flavonoids for their potential health benefits [8, 9, 10].

The objective of the present investigation was to examine the modulatory effect of a phytopolyphenol, apigenin (4′,5,7-trihydroxyflavone) (APG), in human peripheral blood lymphocytes *in vitro*. APG is a flavonoid found widely distributed in the leaves and stems of tropical vegetables and fruits, and it has the following structure.[Fig RRS117F1]

**Fig. 1. RRS117F1:**
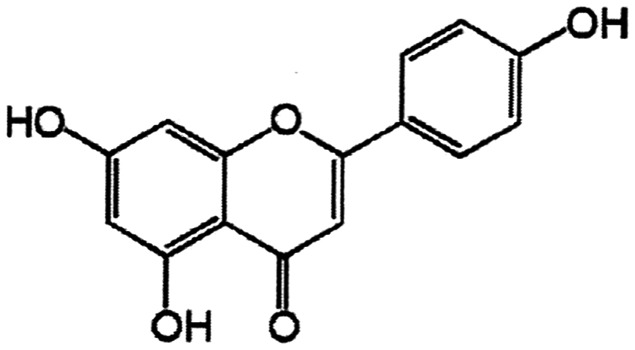
Chemical structure of apigenin (4′5,7-trihydroxyflavone) (APG).

APG has antioxidative, free radical-scavenging, antitumorigenic and antimutagenic properties both *in vivo* and *in vitro* [11, 12, 13, 14]. The present study has been carried out to investigate the effect of APG (10 µg/ml) on gamma ray- (3 Gy) and MMC- (0.02 µg/ml) induced chromosomal damage in human lymphocytes. Gamma-irradiated lymphocytes were post-treated with APG *in vitro* to study its modification of CAs, Sister chromatid exchanges (SCEs) and cytochalasin-B blocked micronuclei (CBMN).

## MATERIALS AND METHODS

### Chemicals

APG, cytochalasin–B, MMC, phytohemagglutinin (PHA-M), Hams F10, fetal bovine serum, demecolcine, L-glutamine, 5 bromodeoxyuridine (BrdU), dimethyl sulfoxide (DMSO) and Hoechst 33258 were purchased from Sigma chemicals Co., St Louis, USA. Giemsa was obtained from Merck Germany.

### Blood donors

Informed consents were obtained from 6 (5 male and 1 female) healthy volunteers in the age group of 26–38 years, with the inclusion criterion of having no history of known exposure to clastogens, smoking, tobacco-chewing, alcohol consumption or drug-taking. Blood samples were drawn from these volunteers under sterile conditions in heparinized vacutainer tubes.

### Irradiation

Aliquots of blood samples were irradiated with ^60^Co gamma rays at a dose rate of 0.77 Gy/min (Teletherapy Machine – Theratron Junior ^60^Co Machine). Blood samples were irradiated with a total dose of 3 Gy. Cultures were set from the irradiated blood samples as per the standard protocol [15].

### Preparation of APG solution

A stock solution of APG (1 mg/ml) in dimethyl sulfoxide (DMSO) was diluted to obtain a concentration of 10 µg/ml in culture. This concentration showed no cytotoxic effect on human lymphocytes and exhibited radioprotective properties [1]. Blood cultures were treated with APG 24 h after the initiation of the cultures.

### Protocol for whole blood lymphocyte culture

Heparinized blood samples were obtained from adult healthy volunteers. Within 24 h of collection of samples, whole blood cultures were initiated by standard procedure. For each culture, 4 ml Hams F10 medium with 200 mM L-glutamine, 0.5 ml fetal bovine serum and 0.1 ml PHA (reconstituted medium) were inoculated with 0.3 ml whole blood. No antibiotics were added to the cultures at any stage. Cultures were incubated at 37°C. Cultures were terminated after 48 h following a final 3 h treatment with demecolcine at a concentration of 0.02 µg/ml. For SCEs, BrdU was added at a final concentration of 10 µg/ml at the initiation of the cultures. The cultures were terminated after 72 h incubation at 37°C in the dark, following a final 3 h treatment with demecolcine at a concentration of 0.02 µg/ml, then harvested using the conventional procedure involving hypotonic KCl treatment, fixation with methanol-acetic acid (3:1) and air drying on chilled wet slides.

### Study design The following sets of experiments were conducted

**Set I** – 0.3 ml of whole blood was inoculated into 5 ml of reconstituted culture medium and the following groups were included:

(a) control, (b) APG 10 µg/ml, 24 h, (c) MMC 0.2 µg/ml, 25 h, (d) APG 10 µg/ml, 24 h + MMC 0.2 µg/ml, 25 h; total duration 72 h (BrdU incorporated at the time of initiation).

**Set II** – 0.3 ml of whole blood was inoculated into 5 ml of reconstituted culture medium for MN analysis and the following groups were included:

(a) control, (b) APG 10 µg/ml, 24 h, (c) MMC 0.2 µg/ml, 25 h, (d) APG 10 µg/ml, 24 h, + MMC 0.2 µg/ml, 25 h; total duration 72 h (cytochalasin B 6 µg/ml added at 24 h).

**Set III** – 0.3 ml of irradiated whole blood was inoculated into 5 ml of reconstituted culture medium for CA analysis and the following groups were included:

(a) control, (b) APG 10 µg/ml, 24 h, (c) ^60^Cobalt γ radiation 3 Gy, 0 h, (d) ^60^Cobalt γ radiation 3 Gy, 0 h + APG 10 µg/ml at 24 h; total duration 48 h (demecolcine added at 45 h).

**Set IV** – 0.3 ml of irradiated whole blood was inoculated into 5 ml of reconstituted culture medium for MN analysis and the following groups were included:

(a) control, (b) APG 10 µg/ml, 24 h, (c) ^60^Cobalt γ radiation 3 Gy, 0 h, (d) ^60^Cobalt γ radiation 3 Gy, 0 h + APG 10 µg/ml at 24 h; total duration 72 h (cytochalasin B 6 µg/ml added at 24 h).

### Lymphocyte culture and slide preparation

#### Culture setup for CA, CBMN and SCE

PHA-stimulated whole blood cultures from unirradiated and irradiated blood samples were set up in a reconstituted medium following the standard procedures as described above for obtaining metaphase chromosomes. APG (10 µg/ml) was added 24 h after the initiation of cultures. Cultures were treated with demecolcine (0.02 µg/ml) at 45 h, incubated further for 3h and treated with 0.02 µg/ml demecolcine, 3 h before the harvest to study the CAs. Air-dried preparations of hypotonically treated, methanol:acetic acid fixed lymphocytes were made, using routine techniques for chromosome analysis, and stained with Giemsa stain 1% in phosphate buffer (Sorensen's buffer), pH 6.8, for 20 min. [15, 16]. The culture protocol followed for CBMN was the same as that described above. Briefly, at 24 h after culture initiation, cytochalasin B was added, resulting in a final concentration of 6 µg/ml in the cultures. APG (10 µg/ml at 24 h) and MMC (0.02 µg/ml at 25 h) were added to the cultures. Cells were harvested at 72 h following a 5 min 0.8% cold KCl treatment and fixation, including 1% formaldehyde in the second fixative. Cells were stained with 1% Giemsa (Merck) in Sorensen's buffer, pH 6.8, for 20 min [17, 18, 19]. The culture protocol followed for SCE analysis was same as described above for obtaining metaphase chromosomes. BrdU (10 µg/ml) was added at the time of initiation of PHA-stimulated whole blood cultures. APG (10 µg/ml) was added at 24 h, and MMC (0.02 µg/ml) at 25 h. Cultures were treated with demecolcine (0.02 µg /ml) at 69 h, and harvested at 72 h.

A modified FPG staining method was applied to obtain harlequin chromosomes. Two-day-old chromosome preparations were stained with Hoechst 33 258 (100 µg/ml in distilled water) for 20 min, rinsed in tap water and mounted under a cover-slip in Sorensen's buffer (M/15, pH 8.0 adjusted with 5% NaOH) and were exposed to 360 nm light from a black ray lamp (distance 2 cm, 20 J/m^2^/s) for 12 min on a slide warming tray at 60°C. Slides rinsed in ice-cold Sorensen's buffer, pH 6.8, followed by rinsing in tap water, were stained with 4% Giemsa (Merck) in Sorensen's buffer pH 6.8 [18, 20, 21]. Harlequin staining of BrdU-substituted chromatids by FPG staining allowed scoring of SCEs in second division mitosis and cell cycle kinetics.

### Cytogenetic analysis

#### CA assays

The stained preparations were examined for unstable CAs [22, 23]. CAs were scored from 100 well-spread metaphases with a minimum of 46 centromeres and 50 metaphases per slide under oil immersion at × 100 magnification.

#### SCE assays

The frequency of SCEs was determined from 50 second-division metaphases, 25 metaphases per slide. Every point of breakage and rejoining was counted as one exchange. An intercalary-exchanged chromatid piece was counted as two exchanges. A terminal exchange was counted as one exchange. The cell proliferation kinetics were estimated as the proliferation rate index (PRI). The PRI was evaluated for 200 metaphases by scoring the number of cells in the first, second, third or subsequent divisions in the FPG-stained slides, using the following formula:

PRI = [M1 + (2 × M2) + (3 × M3) + (4 × M4)]/*n*

where M1 to M4 represent the mitotic cell's 1–4 cell cycle; *n* is the total number of mitotic cells scored [18, 21].

#### CBMN assays

Total numbers of micronucleated cells (MNBN) and total number of MN were determined in 1000 binucleated cells with well-preserved cytoplasm. The nuclear division index (NDI) was determined by scoring the number of mononucleate, binucleate, trinucleate, tetranucleate and more (polynucleate) cells in 1000 viable cells. The nuclear division index (NDI) was calculated as:

NDI = [M1 + (2 × M2) + (3 × M3) + (4 × M4)]/*n*

where M1 to M4 represent the number of cells with 1 to 4 nuclei respectively; *n* is the total number of cells scored [17, 18, 24].

## RESULTS

### CAs

Table 1 shows data obtained in control and gamma-irradiated human lymphocytes cultures with and without APG treatment. CAs exhibiting chromatid breaks, dicentrics, centric rings and acentric fragments were analyzed. It was seen that the level of CAs in cultures treated with APG (10 µg/ml) was comparable to those observed in the control group. It was also observed that the frequency of dicentrics, fragments, total aberrations and total damages per cell in cultures irradiated with radiation (3 Gy) were not significantly different from those irradiated (3 Gy) and then treated with APG (10 μg/ml).

**Table 1.** Effect of APG on the frequency of CAs in human lymphocytes after gamma irradiation

Treatment#	Dicentrics/cell	Total aberrations/cell	Total damages/cell
control^a^	0.00	0.00	0.002
APG 24 h^b^	0.00	0.015	0.015
3 Gy 0 h^c^	0.438	0.693	0.394
3 Gy 0 h + APG 24 h^d^	0.521	0.908	0.462

^a^Control cultures – without any treatment. ^b^APG added 24 h after initiation (PHA stimulation) of cultures. ^c^Whole blood irradiated with 3 Gy and cultures initiated (PHA stimulation). ^d^Whole blood irradiated with 3 Gy and cultures initiated (PHA stimulation), and APG added 24 h after initiation.

The frequency of dicentrics, fragments, total aberrations and total damages per cell was not statistically significantly different in cultures irradiated with3 Gy from those irradiated with 3 Gy then treated with APG (10 µg/ml).

### CBMN

#### MN analysis in irradiated lymphocytes

Table 2 shows the data obtained from control and gamma-irradiated human lymphocytes cultured with and without APG treatment. The control, APG, gamma-irradiated 3 Gy and gamma-irradiated 3 Gy + APG-treated cultures did not show statistically significant difference in the frequency of cytochalasin-B blocked micronuclei.[Fig RRS117F2]

**Fig. 2. RRS117F2:**
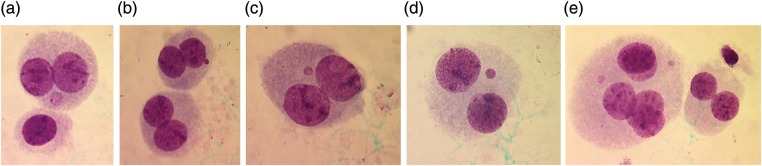
Cytochalasin B-blocked human lymphocytes showing micronucleus. **(a)** A binucleated cell with MN in cytoplasm; **(b)** Two binucleated cells, one cell with MN in cytoplasm: **(c)** and **(d)** Binucleated cells with MN; and **(e)** A binucleated cell and a pentanucleated cell with two MN in the cytoplasm.

**Table 2.** Effect of APG on the frequency of CBMN in human lymphocytes after gamma irradiation

Treatment	MN%
control^a^	1.55 ± 0.2446
APG 24 h^b^	2.35 ± 0.7864
3 Gy 0 h^c^	42.533 ± 2.7357
3 Gy 0 h + APG 24 h^d^	42.533 ± 2.8353

^a^Control cultures – without any treatment. ^b^Apigenin added 24 h after initiation (PHA stimulation) of cultures. ^c^Whole blood irradiated with 3 Gy and cultures initiated (PHA stimulation). ^d^Whole blood irradiated with 3 Gy and cultures initiated (PHA stimulation), and apigenin added 24 h after initiation.

Cultures irradiated with 3 Gy did not show statistically significant difference in the frequency of CBMN when compared with those irradiated (3 Gy) and then treated with APG (10 µg/ml).

#### MN analysis in MMC-treated lymphocytes

The MN frequency in control, APG, MMC, and APG + MMC groups ranged from 1.00–2.60, 0.80–3.40, 2.60–8.00, 2.20–5.80, respectively, and the NDI ranged from 1.48–1.71, 1.25–1.75, 1.44–1.69 and 1.42–1.77, respectively. The data show significant reduction (*P* < 0.01) (first five donors) in the frequency of MMC-induced MN by APG. Cultures treated with APG at 24 h and then with MMC one hour later showed 29.27% to 71.79% inhibition of MN frequency when compared with expected values. Donor no. 6* showed a very high frequency of MN percentage in treated cultures when compared to control.[Fig RRS117F3]

**Fig. 3. RRS117F3:**
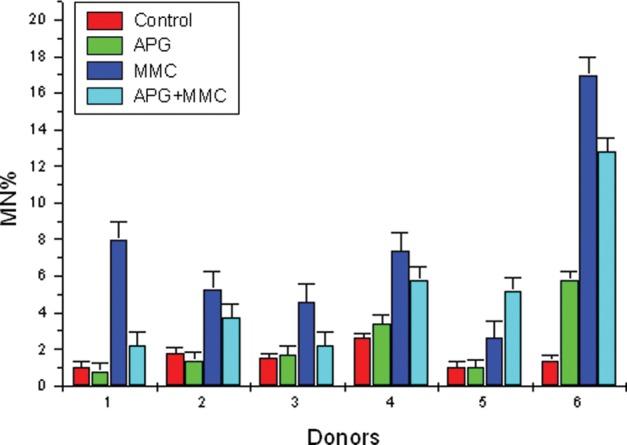
MN frequency analyzed in control, APG, MMC and APG + MMC in human lymphocytes *in vitro* in individual donors.

Table 3 shows the frequency of MN, NDI and percent inhibition in MMC-treated lymphocytes by APG in the culture samples of the 6 donors. The expected and observed MN frequency was determined by adding up values of MN frequency observed in APG-treated and MMC-treated cultures, then subtracting the value of MN frequency observed in control cultures. The MN frequency obtained in APG + MMC-treated cultures were considered as observed values. The expected MN frequency ranged from 4.80–8.20, and the observed MN frequency ranged from 2.20–5.80. The data showed a significant reduction of MN frequency (*P* < 0.01) (first 5 donors).[Fig RRS117F4]

**Fig. 4. RRS117F4:**
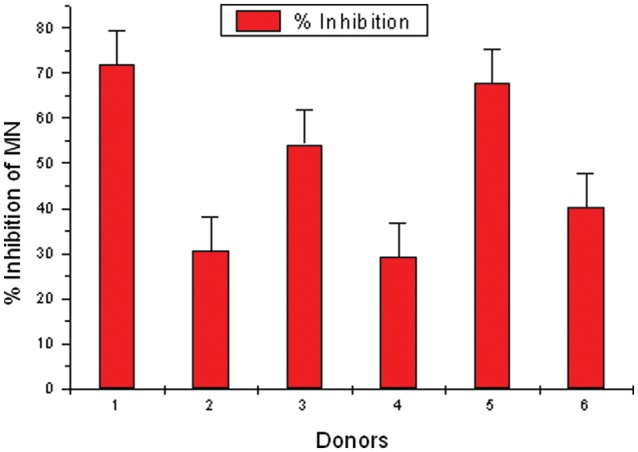
Effect of APG on percent inhibition of MN in human lymphocytes treated with MMC *in vitro*. APG- and MMC-treated lymphocytes showing the inhibition percentage in MN frequency in individual donors. Individual variability is observed in donors.

**Table 3.** Effect of APG on the frequency of MN%, NDI and percent inhibition in human lymphocytes treated with MMC *in vitro*

Donor	control MN%	NDI	APG MN%	NDI	MMC MN%	NDI	APG + MMC MN%	NDI	(b + c) – a = x	X – y = z	z/y	% inhibition
	a		b		c		y (observed)		x (expected)	z		z/x%
1	1.00	1.70	0.80	1.70	8.00	1.60	2.20*	1.71	7.80*	5.60	0.717 949	71.794 9
2	1.80	1.48	1.40	1.46	5.80	1.44	3.75*	1.59	5.40*	1.65	0.305 556	30.555 6
3	1.50	1.71	1.70	1.78	4.60	1.69	2.20*	1.77	4.80*	2.60	0.541 667	54.166 7
4	2.60	1.69	3.40	1.45	7.40	1.49	5.80*	1.45	8.20*	2.40	0.292 683	29.268 3
5	1.00	1.70	1.00	1.25	2.60	1.53	5.20*	1.42	6.80*	4.60	0.676 471	67.647 1
6	1.40	1.69	5.80	1.25	17.00	1.55	12.80**	1.58	21.40	8.60	0.401 869	40.186 9

Group* Expected Observed

Mean 6.6 3.23

Standard deviation 1.476 48 1.585 72

Standard error ± 0.660 3 ± 0.709 15

*n* 5 5

*P < 0.01 (5 donors)

### SCE

Analysis of data revealed an increase in the frequency of SCEs per cell in APG-treated samples compared with the controls. Cultures treated with 10 µg/ml of APG at 24 h, followed by MMC treatment one hour later, showed significant decrease (*P* < 0.01) (5 donors) in the frequency of SCEs when compared with MMC treatment.[Fig RRS117F5][Fig RRS117F6][Fig RRS117F7]

**Fig. 5. RRS117F5:**
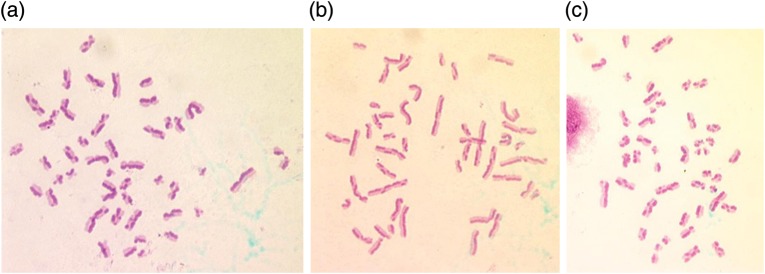
Sister chromatid differentiation based on BrdU incorporation in human lymphocyte chromosomes demonstrated by FPG staining. Second-division metaphase spreads with SCEs **(a, b, c)** APG-treated.

**Fig. 6. RRS117F6:**
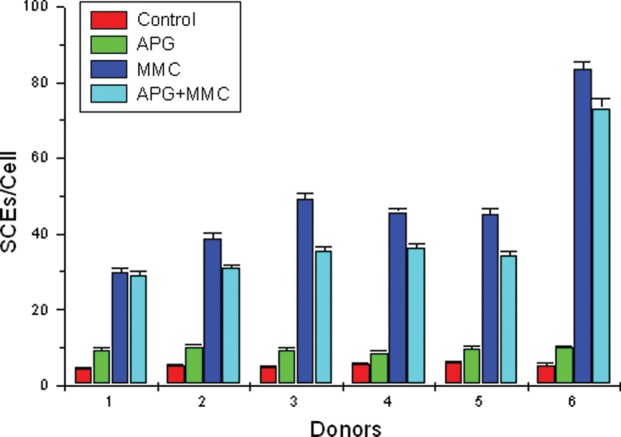
SCE frequency analyzed in control, APG, MMC and APG + MMC in second-division metaphase chromosomes *in vitro* in individual donors.

**Fig. 7. RRS117F7:**
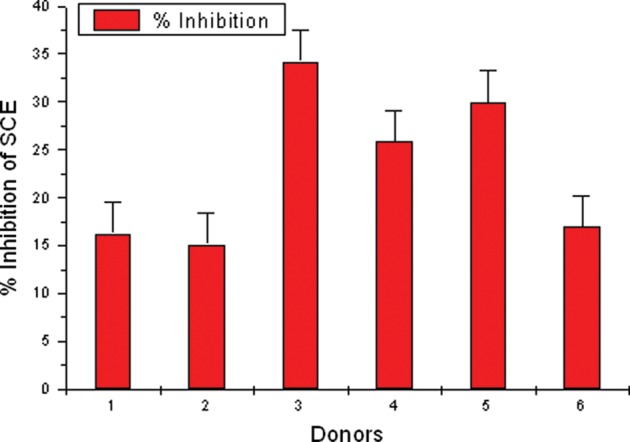
APG and MMC-treated lymphocytes showing the inhibition percentage in SCE frequency in individual donors. Individual variability is observed in donors.

Table 4 shows the frequency of SCEs, PRI and percent inhibition by APG in MMC-treated lymphocytes in the 6 donors. The SCE frequency in control, APG, MMC, APG + MMC-treated groups, ranged from 4.56–6.12, 8.64–10.18, 29.88–49.32 and 29.00–37.02, respectively. Further, the PRI ranged from 2.20–2.57, 1.88–2.13, 1.78–2.40 and 1.68–2.01, respectively. Cultures treated with APG at 24 h and MMC one hour later showed 15.13% to 34.21% inhibition of SCEs. The data show significant reduction of SCE frequency (*P* < 0.01) (first 5 donors). Donor no. 6** showed a very high frequency of SCEs per cell in treated cultures when compared with the control.

**Table 4.** Effect of APG on the frequency of SCEs per cell, range of SCE, PRI and percent inhibition in human lymphocytes treated *in vitro* with MMC

Donor	Endpoint	Control	APG	MMC	APG + MMC	(b + c) – a = x	x – y = z	% inhibition
		a	b	c	y Observed	Expected		z/x*100
1	SCE	4.56 ± 0.344	9.30 ± 0.500	29.88 ± 0.928	29.00 ± 1.106	34.62*	5.62	16.23
	Range	1–10	4–20	16–48	6–45			
	PRI	2.50	2.13	2.40	2.01			
2	SCE	5.34 ± 0.371	10.18 ± 0.484	38.78 ± 1.344	37.02 ± 1.030	43.62*	6.60	15.13
	Range	1–12	3–19	18–64	22–58			
	PRI	2.57	1.88	1.91	1.74			
3	SCE	4.84 ± 0.367	9.36 ± 0.534	49.32 ± 1.546	35.42 ± 1.143	53.84*	18.42	34.21
	Range	0–10	3–22	31–71	18–53			
	PRI	2.20	2.01	2.07	1.68			
4	SCE	5.52 ± 0.406	8.64 ± 0.531	45.78 ± 0.957	36.26 ± 1.088	48.90*	12.64	25.85
	Range	1–17	3–18	34–63	22–60			
	PRI	2.22	2.05	2.03	1.77			
5	SCE	6.12 ± 0.408	9.68 ± 0.522	45.30 ± 1.314	34.24 ± 0.999	48.86*	14.62	29.92
	Range	0–13	3–18	32–70	23–58			
	PRI	2.33	2.09	1.78	1.70			
6	SCE	5.32 ± 0.453	10.02 ± 0.519	83.50 ± 2.137	73.26 ± 2.584	88.20**	14.94	16.94
	Range	2–17	2–21	58–110	43–108			
	PRI	2.14	2.07	1.38	1.68			

50 second-division metaphases scored for SCEs. 200 cells scored to determine PRI, *P < 0.01 (first 5 donors).

## DISCUSSION

In this investigation, we evaluated the modulatory effects of APG on the chromosomal damage induced by ionizing radiation (3 Gy) and an anti-neoplastic agent, MMC, using human peripheral lymphocytes. We used CA, SCE and CBMN formation as the end points. Our data indicated that the control (untreated) lymphocytes, did not exhibit any type of chromosomal damage, measured as CA, SCE or CBMN formation.

We observed that ionizing radiation (3 Gy) alone caused chromosomal damage, as measured by CAs, such as dicentrics, centric rings and fragment formation. Lymphocytes treated with APG (10 µg/ml) 24 h after irradiation with 3 Gy did not reduce the extent of chromosomal damage in terms of CAs in lymphocytes. Further, APG *per se* did not cause CAs over and above those caused by 3 Gy radiations**.** Rithidech *et al*. [1] studied the frequency of MN in human lymphocytes induced by various concentrations of APG alone, and in combination with a single dose of 2 Gy ^137^Cs gamma rays. Their study showed that APG at concentrations between 2.5 µg/ml and 10 µg/ml had no significant effect on the induction of chromosomal damage in human lymphocytes exposed *in vitro*. However, a significant increase in the frequency of chromosomal damage was observed at higher concentrations (> 25 µg/ml). They also reported that the presence of APG at all the concentrations (0, 2.5, 5, 10 and 25 µg/ml) during irradiation reduced the frequency of chromosomal damage in a concentration-dependent manner when compared with the control cells that were irradiated in the absence of APG.

The presence of APG during irradiation seems to be an important factor, in order to have any radioprotective effect on human chromosomes. Thus, Rithidech *et al*. [1] have shown that treatment with APG, followed by washing of the cells before irradiation with 2 Gy ^137^Cs gamma rays, failed to reduce the frequency of chromosomal damage. Their study indicated that the active presence of APG during irradiation is required for a radioprotective effect. We have used APG to modulate the effects of 3 Gy ionizing radiation, as a post-irradiation treatment. Many researchers have reported the effects of APG as a pre-treatment for radiation-induced chromosomal damage [1]. We used APG at a concentration of 10 µg/ml, since earlier studies [1] showed that at this concentration APG did not exhibit genotoxicity. We found that APG used at an optimal concentration of 10 µg/ml 24 h post-radiation treatment, had no protective effect on human lymphocyte chromosomal damage, measured as CA and CBMN formation. There are not many reports on the use of a radioprotective agent, such as APG, used as a post-radiation treatment. In our study, we observed that the frequency of dicentrics, fragments, total aberrations and total damages/cell did not show any significant difference when comparing 3 Gy 0 h with 3 Gy 0 h + APG 24 h. This indicates the non-genotoxic nature of APG. The observed cytoprotective effect of APG may be due to the possible scavenging of radiation-induced electrophiles/nucleophiles, or by modulating the DNA repair system in human peripheral blood lymphocytes [1].

In our study, the whole blood samples were first irradiated and cultured, and APG was added at 24 h after initiation of culture, which may have produced no significant result in the irradiated blood samples. It has been reported that APG is absorbed and metabolized by human cells after intake, and its half-life is reported to be about 12 h, as measured by urinary excretion [25]. Lack of the active presence of APG during irradiation in our study may explain the failure of APG to offer any radioprotective effect against human lymphocyte chromosomal damage.

Our study also provides evidence of the modulatory effects of APG on the induction of MN and SCEs in human lymphocytes treated *in vitro* with MMC. The results also confirm that 10 µg/ml of APG is non-toxic and optimum for experimental studies. The lymphocytes were treated with APG 24 h after the initiation of the cultures. The results show a protective effect of APG in human lymphocytes, reflected in the reduction of SCE and CBMN formation. One donor showed very high values of MN%, and SCEs/cell in treated cultures when compared with control. This property may be attributed to individual variation and genetic makeup, or hidden genetic instability. However, the NDI and PRI of this donor were comparable with the other 5 donors, which indicated that in all the 6 donors, the cell cycle kinetics were not inhibited.

The SCE assay in human peripheral lymphocytes is widely used to detect occupational and environmental exposures to genotoxic compounds because it has been shown that is a highly sensitive parameter for evaluating human exposure to mutagenic and carcinogenic agents [26, 27]. The antigenotoxic effect of APG has been reported using MMC on mouse bone marrow cells and measuring SCEs and CAs [28]. In the present study, a significant decrease in SCEs and CBMN was observed, suggesting a protective role of APG against the genotoxicity of MMC. MMC is an anti-tumor, antibiotic compound that has a range of genotoxic effects, including the inhibition of DNA synthesis, mutagenesis and clastogenesis. It is a direct-acting clastogen requiring only intracellular reductive activation to initiate its potent DNA cross-linking action [29, 30]. A study conducted by Snyder *et al*. [31] also showed clastogenic activity of APG at 100 µM in Chinese hamster V79 cells, and proposed that the clastogenic activity of APG was due to its ability to intercalate DNA molecules. Genotoxic effects of anti-cancer drugs in non-tumor cells are of special significance as they may induce secondary tumors in cancer patients. Furthermore, the mutagenic and carcinogenic effects of antineoplastic agents on the health-care persons handling these drugs also need to be considered carefully [32]. It is quite possible that the uptake of complex, plant-derived mixtures may modulate the genotoxicity of anti-cancer drugs, and thus may reduced the chances of developing secondary tumors in cancer patients. The genotoxic damage caused by flavonoids at higher concentrations is considered to be due to DNA intercalation, poisoning of DNA topoisomerase II, generation of reactive metabolites and inhibition of key enzymes. Utilization of anti-carcinogenic nutrients could play a vital role in protecting those exposed to chemotherapeutic agents [32, 33, 34, 35].

Several *in vitro* studies have shown that when antioxidants such as ascorbate, vitamin A, and their metabolites are combined with chemotherapeutic drugs, [21] they can enhance the growth inhibitory effects of most of the currently used chemotherapeutic agents on selected cancer types [36, 37, 38]. Because of the aforementioned differences in normal versus cancer cells, flavonoid complementary therapy can protect normal tissues from the adverse effects of chemotherapeutic agents, without negating the therapeutic efficiency. Lower doses of chemotherapeutic agents, combined with selected antioxidants, could be used to obtain the same killing power as higher doses of chemotherapeutic agents. Unlike using higher doses of chemotherapeutic agents, the enhanced efficiency mixture would be expected to reduce the complications associated with the chemotherapeutic agents [37]. Gupta *et al*. [39] demonstrated that oral administration of 20 and 50 µg/day APG to mice with prostate cancer xenografts can significantly inhibit tumor growth but without any apparent signs of toxicity. Reports also showed that APG has no mutagenic activity and is capable of selectively inhibiting cell growth and inducing apoptosis in cancer cells without affecting normal cells.

The exact mechanism for the observed modulation and genotoxic effects of APG is not clear, but oxidative stress due to the formation of reactive oxygen species (ROS), mitochondrial dysfunction, transformation to reactive metabolites [40, 41, 42], DNA intercalation and inhibition of DNA topoisomerase II [43, 44, 45] may play crucial roles. It may act as a mutagen pro-oxidant that generates free radicals, or as an inhibitor of key enzymes involved in hormone metabolism, to produce clastogenic effects depending upon the physiological state. More research on the toxicological properties of flavonoids as “substitute medicine” needs to be carried out in order to determine adverse health effects upon routine intake.

## CONCLUSION

Co-treatment or post-treatment with APG has modulatory effects on SCEs and CBMN in human lymphocytes treated *in vitro* with MMC. The present study also indicates that post-radiation treatment with APG does not offer radioprotection; pre-treatment with APG is essential before irradiation for a radioprotective effect on cells.

## FUNDING

This work was funded by the Department of Atomic Energy.
